# Temporal Theta Power is Related to Age and Working Memory Functioning: Putative Biomarkers for Detecting Non-Salient Pathological Brain States in Older People

**DOI:** 10.1192/bjo.2025.10176

**Published:** 2025-06-20

**Authors:** Oded Meiron

**Affiliations:** Bar-Ilan University, Ramat Gan, Israel

## Abstract

**Aims:** In adult humans, WM-related EEG theta dynamics are hypothesized to reflect involvement of prefrontal-hippocampal theta-rhythms in the integration of working-memory associations into unitary coherent memory representations accessible for selection during memory retrieval. Therefore, medial temporal lobe (MTL) theta oscillations are hypothesized to drive the retention of novel verbal WM associations in humans. Several studies in adult samples reported an increase in theta activity during the WM retention delay. However, the impact of ageing on fronto-temporal power during verbal WM tasks (associated with MTL network plasticity), and on WM performance differences between younger age groups versus older people needs to be further examined. Thus, we aimed to show a significant relationship between theta activity and WM across the lifespan, and to suggest novel diagnostic markers sensitive to early WM memory impairment associated with ageing and prodromal stages of Alzheimer’s disease (AD). Thus, increased theta power, which is required to maintain verbal information under different WM loads is predicted to be lower in older adults versus younger adults, specifically, under lateral fronto-temporal-parietal electrode locations, and during verbal WM retention intervals.

**Methods:** The current investigation examined EEG theta power dynamics in older people (ages 55–84) versus younger people (ages 18–36 years) during verbal working memory task intervals at lateral temporal cortex locations and frontal cortex locations according to the international EEG 10–20 system. All participant were in healthy condition without psychiatric or neurological history.

**Results:** Age was significantly related to verbal WM performance (accuracy and RTs) and to right temporal theta power during WM retention intervals. In accordance, as we age, our right temporal theta activity decreases during WM retention intervals. Importantly, mean theta power during WM retention periods significantly distinguished the young sample from the older sample. All other frontal or parietal theta activity was not related to age, and the effects of age were only detected during the retention intervals occurring during the verbal WM task.

**Conclusion:** If investigated and validated further in MCI and AD patients, excessive left-temporal power in older people with WM impairments observed in mild cognitive impairment (MCI) patients versus healthy controls may represent insufficient neural excitability in global frontal-parietal-MTL networks to sustain verbal memory storage and retrieval functions in aging individuals. Thus, we suggest that future studies on noting left-prefrontal excessive frontal alpha and left-temporal theta activity during routine medical procedures in older people as a biomarker of white matter deterioration (e.g., related to impairments in long-range interhemispheric connectivity) within these networks, also known to precede Alzheimer’s disease (AD). Replicating these findings in larger elderly populations versus AD patients longitudinally, may facilitate EEG-based early detection of AD, as well as facilitate early preventive noninvasive brain-stimulation interventions targeting the right temporal cortex to enhance verbal WM functioning in older people displaying early MCI symptoms, and possibly delay the onset of more pervasive dementia (AD) symptoms.

